# The Early Impacts of the COVID-19 Pandemic on Mental Health Facilities and Psychiatric Professionals

**DOI:** 10.3390/ijerph18158034

**Published:** 2021-07-29

**Authors:** Jade Gourret Baumgart, Hélène Kane, Wissam El-Hage, Jocelyn Deloyer, Christine Maes, Marie-Clotilde Lebas, Donatella Marazziti, Johannes Thome, Laurence Fond-Harmant, Frédéric Denis

**Affiliations:** 1 EA 75-05 Éducation, Éthique, Santé (EES), Faculté de Médecine, Université François-Rabelais, 37020 Tours, France; helene.kane@univ-tours.fr (H.K.); frederic.denis@univ-tours.fr (F.D.); 2 CIC 1415, U 1253 iBrain, Institut National de la Santé et de la Recherche Médicale (INSERM), Centre d’Investigation Clinique, Centre Hospitalier Régional Universitaire (CHRU), 37000 Tours, France; wissam.elhage@univ-tours.fr; 3 Centre Neuro Psychiatrique St-Martin (CNP), 5100 Namur, Belgium; jocelyn.deloyer@saintmartin.ofc.be (J.D.); christine.maes@saintmartin.ofc.be (C.M.); 4 Département des Sciences de la Santé Publique et de la Motricité, Haute Ecole de la Province de Namur (HEPN), 5000 Namur, Belgium; marie-clotilde.lebas@hepn.province.namur.be; 5 Department of Clinical and Experimental Medicine, Section of Psychiatry, University of Pisa, 56126 Pisa, Italy; dmarazzi@psico.med.unipi.it; 6 UniCamillus, Saint Camillus International University of Health and Medical Sciences, 00131 Roma, Italy; 7 Department of Psychiatry, University of Rostock, 18055 Rostock, Germany; Johannes.Thome@med.uni-rostock.de; 8 Agence de Coopération Scientifique Europe-Afrique (ACSEA), L-2010 Luxembourg, Luxembourg; fond.harmant@gmail.com; 9 UR 3412 Laboratoire Education et Pratiques en Santé, Université Sorbonne Paris Nord, 93017 Bobigny, France

**Keywords:** psychiatry, mental health, mental health facilities, mental health professionals, COVID-19, SARS-CoV-2, epidemic, pandemic, systematic review

## Abstract

(1) Background: While in many countries, the psychiatric and mental health sectors had been in crisis for years, the onset of a novel coronavirus pandemic impacted their structures, organizations, and professionals worldwide. (2) Methods: To document the early impacts of the COVID-19 health crisis on psychiatry and mental health sectors, a systematic review of the international literature published in 2020 was conducted in PubMed (MEDLINE), Cairn.info, and SantéPsy (Ascodocpsy) databases. (3) Results: After applying inclusion and exclusion criteria, 72 articles from scientific journals were selected, including papers documenting the early impact of the COVID-19 pandemic on the organization of psychiatric care delivery, work processes in psychiatry and mental health units, and personal experiences of mental health professionals. This review identified the contributions aimed at preventing the onset of mental disorders in the early stages of the health crisis. It lists the organizational changes that have been implemented in the first place to ensure continuity of psychiatric care while reducing the risk of SARS-CoV-2 transmission. It questions the evolution of the rights and duties of mental health professionals in the first months of the pandemic. (4) Discussion and conclusions: Although this literature review exclusively documented the early impacts of the COVID-19 health crisis, it is of significant interest, as it pictures the unprecedent situation in which psychiatry and mental health care professionals found themselves in the first stages of the pandemic. This work is a preliminary step of a study to be conducted with mental health professionals on an international scale—the Psy-GIPO2C project—based on more than 15 group interviews, 30 individual interviews, and 2000 questionnaires. The final aim of this study is to formulate concrete recommendations for decision-makers to improve work in psychiatry and mental health.

## 1. Introduction

Since late 2019, the world has been impacted by the outset and spread of a new coronavirus, SARS-CoV-2, responsible for the COVID-19 coronavirus disease. The World Health Organization declared the outbreak a Public Health Emergency of International Concern on 30 January 2020, and a pandemic on 11 March 2020.

The health crisis accompanying this pandemic has been placing an unprecedented burden on psychiatry and mental health care systems, many of which had already been straining for years [1,2]. A large number of countries—including Australia, France, Ireland, and Scotland—are struggling to provide those sectors with the resources they need to deliver care under the right conditions and to recruit staff [3,4].

The COVID-19 health crisis has led health facilities, institutions, and professionals to adapt their organization as well as their practices. A series of measures have been deployed in the early stages of the health crisis to ensure continuity of care and meet the new needs generated by the crisis [5,6] while reducing the risk of SARS-CoV-2 transmission. In response to this situation, the use of digital technologies, and in particular those of telemedicine tools have grown up to an unprecedented scale. In this context, psychiatric and mental health professionals have had to face several issues: They have had to secure continuity of care for both in- and outpatients while following everyday preventive actions, as well as to deal with the population’s demand for care in this unprecedented and anxiety-inducing situation.

Experience from previous outbreaks (SARS, MERS, Ebola, etc.) has shown that epidemic crisis often leads to serious and lasting consequences for the mental health of populations [7,8,9]. In addition to people whose mental disorders potentially worsen, outbreaks tend to cause psychological reactions in the general population ranging from moderate to excessive anxiety and panic [7]. People who have lost a loved one, those who have narrowly escaped death, and their relatives. In this context of the COVID-19 health crisis, frontline health professional, and health professionals in general, are particularly at risk of developing symptoms of burnout, chronic mental illness as depression, or post-traumatic stress disorder [8,10,11,12]. Psychiatry therefore has a crucial role to play in limiting the onset and aggravation of mental disorders, including in regard with health professionals [13,14].

In such an emergency context, first adjustments were made—some of which were more or less anticipated, even improvised—to cope with the situation and ensure continuity of care. Remote consultations have been set up in psychiatry as well as in mixed psychiatric/COVID units and areas have been created in psychiatric structures, for example. These early adaptations nonetheless raise many questions in the specific field of mental health care.

Through this literature review, we proposed:

- First, to examine the early impact of the COVID-19 pandemic on the organization of psychiatric care and on the management of people suffering from severe or moderate psychological disorders in mental health care systems.
- Second, to assess the first impact of the current crisis on both the working conditions and the mental health of psychiatric professionals.
- Third, to analyze reorganizations and innovative practices implemented in psychiatric care settings or by mental health professionals at the beginning of the health crisis.

## 2. Materials and Methods

The present international systematic review of the literature was conducted following the PRISMA guidelines [15].

After an exploratory search on Google Scholar, the literature search was carried out in three databases: PubMed (MEDLINE), Cairn.info, and SantéPsy (Ascodocpsy). PubMed (MEDLINE) is a database that is traditionally used for literature reviews and allows the collection of English-language publications. To also collect publications in French—as the study is conducted by a French research team—the generalist database cairn.info was also chosen, as well as the SantéPsy database (Ascodocpsy), which is dedicated to resources related to psychiatry and mental health.

The search terms (Table 1) were defined by articulating keywords—previously selected from dictionaries of synonyms and thesauri—and using the Boolean operators “AND”/“OR”. They were constructed with the aim of collecting material that provides specific answers to the issues raised in the study.

The inclusion criteria allowed to select references published from 1 January to 31 December 2020, in English or French, dealing with the impact of the COVID-19 pandemic on the organization of care delivery and work processes in psychiatry and mental health care, as well as references documenting personal experiences of mental health professionals. Articles of any type were included, except editorials.

The exclusion criteria eliminated references dealing with the impact of the COVID-19 pandemic on the mental health of the general population or on the mental health of health professionals in general, as well as references related to the neurological and psychiatric effects of SARS-CoV-2, and the psychological effects and neuropsychiatric sequelae of the health measures that have been adopted and implemented. References focusing exclusively on the use of telepsychiatry were not included, and neither were those dealing only with the impact of the COVID-19 pandemic on the training of future mental health professionals, since it seemed more relevant to address these topics in more depth in separate literature reviews.

**Figure 1:**
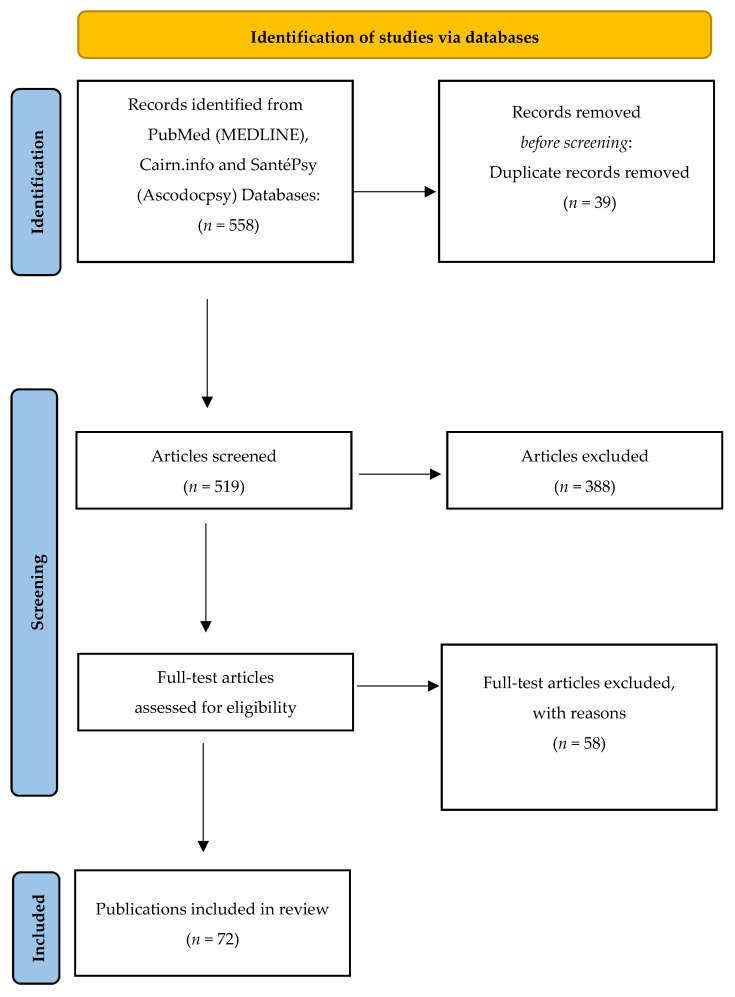
Flow chart.

The research yielded 558 documents, including 39 duplicates that were deleted. By applying inclusion and exclusion criteria, first on titles and abstracts, and then on full texts, the first two authors (Gourret Baumgart J and Kane H, respectively a study engineer and research engineer in the Psy-GIPO2C project), performed the search and agreed to select 72 references (Figure 1). Each of these documents was carefully read (Table 2) and analyzed (Table 3).

## 3. Results

The first observation is that many articles were published in a relatively short period of time. Over the three-month period of this literature review, the number of publications increased rapidly, from 448 articles on 1 November 2020 to 501 on 1 December 2020, and then to 558 articles on 1 January 2021.

Geographically speaking, most of the selected articles are about, or their authors are affiliated with, European countries [30]. Next, many articles follows related to America [17], and then some to Asia (8). Finally, a few articles concern Oceania (3) and Africa (1). Some publications are referred to as “international” in that they deal with, or their authors are affiliated to, several countries (13).

As for the type of articles, most are feedback from personal or field experience written by psychiatric and mental health professionals (38), followed by studies (17), and then literature reviews (13), either narrative or systematic. Finally, a few selected articles are reflections focused on the issue we are addressing (4).

Three major themes emerged:

1. Many contributions have been aimed at preventing the onset of mental health disorders in the context of a COVID-19 health crisis;
2. Reorganizations were implemented in psychiatric care facilities and units to reduce the risk of SARS-CoV-2 transmission;
3. The rights and duties of mental health professionals regarding involuntary treatment have evolved in the context of the COVID-19 pandemic.

### 3.1. Preventing the Onset of Mental Health Disorders in the Context of a Health Crisis

#### 3.1.1. An Increasing Role of Care towards the General Population

In the context of the COVID-19 pandemic, mental health professionals have played a key role in the general population [16,17,18,19,20,60,61]. To mitigate the impact of the health crisis, a large number of programs, aimed at the general population, and focused on the prevention of mental disorders and the promotion of wellbeing, have been implemented, throughout the world. Most of these programs included the use of the phone or the computer [16,17,18,19,20,60]. Mental health professionals have also been involved in providing specific responses to people who found themselves in vulnerable situations—particularly those in relation to containment measures—that could lead to psychological distress or even mental disorders, among which those associated with substance use [20]. In that respect, direct interventions have been carried out towards people living in poverty, homeless people, or victims of domestic abuse and violence [20].

#### 3.1.2. An Expanding Role of Support towards Other Health Professionals

In this context, mental health professionals also provide support to other health professionals, especially to the so-called “front-line health professionals”, who are involved in the care of COVID-19 patients and exposed to stressful situations [44,45,46,47,71]. This is the case of workers in consultation-liaison psychiatry teams, who were already providing support to other healthcare staff members through stress and burnout prevention programs [45]. Programs and plans have been developed specifically to provide psychological or even therapeutic support to health professionals [44,46,47,71]. One of these programs, developed in Turkey, offers support services to the children of health care workers, with the concomitant aim of helping the latter [71]. Whether in existing programs implemented in the context of the COVID-19 health crisis [45] or in those created specifically to address it [44,46,47,71], common logic is at work: The purpose of these interventions is to improve the wellbeing of health care workers and build their resilience so that they can continue to work in the conditions that their duties require and thus contribute to maintaining the overall functioning of healthcare systems [47].

#### 3.1.3. Interventions in Other Work Contexts to Support Fellow Health Professionals

Mental health professionals also intervened—including using digital technologies—in psychiatric and mental health environments where they are not used to work, or in units dedicated to COVID-19 positive patients who are hospitalized under anxiety-provoking conditions of isolation [48,49,62]. In the United States, for example, psychiatrists and psychiatry students assisted palliative care teams deployed to COVID-19 patients in critical situations [48,49]. The mental health professionals whose tasks were thereby reassigned have had to adapt to this new environment and working conditions [21].

### 3.2. Reorganizing Psychiatric Facilities to Reduce the Risk of SARS-CoV-2 Transmission

#### 3.2.1. Addressing Psychiatric Patients’ Higher Risk of Infection

Due to frequent comorbidities and a weakened immune system, people with mental disorders are at increased risk of infections and severe diseases—especially infectious and pulmonary ones [13,41,54,66]. Even some symptoms of mental illness also often make it harder for them to practice everyday preventive actions [39,41]. Actually, there is frequently a dichotomy between physical and mental health care, which partly explains the importance of somatic comorbidities in these populations [9,21,66]. Psychiatric hospitals rarely have somatic care units, and within this context, psychiatric care professionals have had to adapt to impose preventive measures and organize care for both patients with symptomatic and asymptomatic forms of the virus [13].

#### 3.2.2. Organizing to Prevent Clusters

Since they gather many people in a limited space, psychiatric hospitals are particularly at risk of becoming transmission clusters [7,8,41,59,66], especially since community life and shared activities such as eating together, therapy groups, physical activities, and sports are important parts of therapeutic plans [13,21,41,54,66].

#### 3.2.3. Implementing Multiple Adaptations in Psychiatric Facilities

A significant number of adjustments have been made in psychiatric hospitals to mitigate the risk of SARS-CoV-2 transmission within while ensuring continuity of psychiatric care [9,13,20,21,22,23,25,26,27,29,30,31,35,36,38,39,42,43,49,52,54,55,58,59,63,64,66,67,68,77,79,80].

They have included preventive measures such as maintaining physical distance and wearing appropriate masks and other adequate personal protective equipment (PPE), as well as cleaning and disinfecting protocols for equipment and premises. Spaces have also been restructured: Many facilities set up areas or units dedicated to patients with suspected or confirmed COVID-19, or areas for screening procedures such as assessing body temperature and testing [9,21,22,26,29,36,54,63,64,66]. In addition, procedures, markings, and signage were set up to guide traffic and provide advice on preventive actions [9,21,26,54,64]. Many facilities also banned in-person visits to patients [21,26,36,59].

Some hospitals have organized regular team meetings to brief mental health staff members [26], while others have set up training for psychiatric staff in the care of patients with COVID-19 or in the management of the risk of SARS-CoV-2 transmission [9,29,64,65]. This should allow both a better quality of care and a better quality of work life, since a Chinese study showed that those who have received relevant training would be less afraid of being infected and of infecting their relatives [65].

In addition to these measures, a strategy common to most psychiatric facilities consisted of scaling down their levels of activity, by reducing or even cease outpatient and day-hospital activities, or by cutting down full inpatient admissions as well as the number of available and occupied beds [20,28,59]. As activity declined, more or less formal and formalized approaches led to prioritizing care and identifying the most vulnerable patients [25,26,29]. Such a decrease, reflecting a reduction of inpatients numbers in those facilities, was generally associated with a reduction in the number of staff [21,56].

Another strategy common to most psychiatric hospitals was for professionals to use digital tools in order to ensure continuity of care while reducing the risk of SARS-CoV-2 transmission [20,22,25,26,28,30,35,39,42,43,48,54,59,63,69,76]. Whereas the use of telepsychiatry was marginal before the health crisis due to the COVID-19 pandemic [24,52], the use of videoconferencing tools—including Skype, WhatsApp, and Zoom—has grown considerably worldwide—in America, Europe [43], the Middle East [76], Asia [63], etc. Actually, this unprecedented extension of telepsychiatry was facilitated by a general alleviation of constraints on its use and reimbursement [58]. In the context of the COVID-19 pandemic, videoconferencing technologies have mainly been used to monitor patients who were usually followed on an outpatient basis and to maintain group therapies, but also to conduct general staff meetings and meetings between professionals [59]. In some cases, the shift from face-to-face to remote communication—through the use of videoconferencing technologies—has been implemented in conjunction with teleworking [52,68]. The mental health professionals who transitioned to telework have had to adapt to these new home-working settings [52,68].

#### 3.2.4. Setting Up Extra-Psychiatric Care Structures

The adaptations implemented in private practices to reduce the risk of SARS-CoV-2 transmission show that the measures and strategies adopted were relatively the same as those of psychiatric care facilities, implying a reduced activity as well as the use of telepsychiatry and of home-based work [24,58,69]. Community mental health facilities have also been forced to adapt their practices to the context [6,27,56,75], replacing as much as possible home visits with phone or videoconferencing consultations, and working from home to avoid sharing an office with other staff [6,56].

In addition to the challenges inherent in maintaining continuity of care within psychiatric wards, mental health professionals have been impacted by the restrictions imposed in the physical care units they work with [35,59,74]. They have also faced challenges in the prescription and delivery of psychiatric medication because of remote follow-up and possible interactions with COVID-19 drugs [53,56,73,75].

### 3.3. Evolving Regulations on Mental Health Professionals’ Rights and Duties in the Context of the COVID-19 Pandemic

#### 3.3.1. Questionable Evolution in the Legal Framework for the Practice of Mental Health Professionals

In the context of the COVID-19 pandemic, national regulations concerning psychiatric care—and more specifically remote psychiatric care and involuntary psychiatric treatment—have been reshaped in several countries [32,52,58,78,81].

In particular, in many countries—including Canada and the USA—the use of telepsychiatry has been facilitated by a general alleviation of constraints on both its use and reimbursement [52,58].

Regulatory changes have also been implemented in the area of involuntary psychiatric treatment. For example, in Germany, the law hitherto in force provided that any individual with mental disorders who is subject to involuntary admission or restraints should be heard personally by a judge, who then had to render a decision authorizing to apply those measures. In the context of the COVID-19 pandemic, it has been allowed to avoid legal hearings in such cases, on the grounds of “*protecting health care and judicial personnel as well as patients*” [78]. Similarly, in Ireland, the law providing a framework for the state of emergency changed the procedures for involuntary admissions to psychiatric hospitals [32]. Until then, the legislation provided a specific procedure whereby a psychiatric assessment report had to be issued by an independent psychiatrist, who had to be recognized as such; this report was intended to be a working basis for a court composed of a consultant psychiatrist, a lawyer, and a lay representative to render a decision regarding the patient’s treatment. In the context of the COVID-19 pandemic, it has been allowed that the psychiatric report would be drawn up by a consultant psychiatrist who did not have to be recognized as an independent expert—with the psychiatric assessment being completed in person or using digital technologies; and that the court could be composed only of a lawyer—with hearings being held by telephone or videoconferencing.

#### 3.3.2. A Lack of Guidance on Professional Practices for Mental Health Workers

While regulations concerning the use of telepsychiatry and involuntary admissions and treatment have been reshaped to fit with the new context, no provisions were adopted—at least initially—to address situations in which a patient refuses to be tested for SARS-CoV-2, thus leaving a legal vacuum. Moreover, mental health care facilities have tried to guide their staff, but psychiatric professionals had little or no recommendations to guide them in dealing with new problematic situations such as involuntary testing [55,81]. Paradoxically, this lack of guidance on some professional practices has been accompanied by the fact that mental health professionals found themselves confused before the “tsunami of information on COVID-19”, which usually took the form of e-mails that were too detailed, unequivocal, or even contradictory [26]. Their personal and professional ethics have been challenged in the face of contradictory orders and incessant changes in policies designed to manage the health crisis [16]

## 4. Discussion

The present literature review highlights that, during the first months of the COVID-19 pandemic, all those adaptations implemented to fit the unprecedented context—including new assignments, reorganizations, innovations in psychiatric care, and regulatory changes for professionals—have had a considerable impact on mental health professionals globally, not only on their working conditions and well-being at work, but also on their personal and professional ethics [16,21,29,34,77]. The challenge was to ensure continuity of psychiatric care while reducing the risk of SARS-CoV-2 transmission; professionals had to get used to new working environments, procedures, and technologies while managing their own stress induced by the health crisis [34]. In this context, making it difficult or even impossible to take time off work, the risk of burnout has become more important for professionals who have kept their activity [13,21]. The fear of being infected with SARS-CoV-2 in the workplace has generally been lower among professionals working in the field of psychiatry than among those working in ambulances, eldercare, and childcare [37]; however, mental health professionals working in geriatric psychiatry mentioned, for instance, their concern about the risk of unintentionally infecting their patients, particularly because of the existence of asymptomatic forms of the disease [34].

This work also highlights that the unprecedented organizational adaptations implemented in psychiatric care facilities in the first months of the COVID-19 pandemic are relatively comparable from one working environment to the other, whether in general psychiatric units such as psychiatric emergency departments, or in specialized psychiatric units such as child psychiatry [29,35,43,79], geriatric psychiatry [36,42], addiction units [30,49], and even in prison psychiatry and forensic psychiatry units [31,33,53,57]. While early adaptations are globally the same from one work context and one country to another, organizational responses came faster in some countries than in others. In Taiwan, for instance, systems that had already been set up in the context of the SARS epidemic were deployed again [9].

Articles from this literature reviews point out that the use of mental health services decreased during previous epidemics (Ebola, MERS, SARS, etc.), but that the need for psychiatric and mental health care increased afterwards [28,51,72]. They highlight the need to prepare for this “post-pandemic mental health tsunami” [5,7,8,51].

### Strengths and Limitations

This systematic review has not been registered. Furthermore, as it includes articles published in 2020 alone, it only documents the early impacts of the COVID-19 health crisis, in the first stages of the pandemic. Plus, it should be noted that many articles included in this literature review were published as early as April and May 2020, and that they are mainly based on a rather weak scientific methodology. This reveals a readiness to publish as well as a lack of maturity on some issues, since carrying out studies with a strong scientific methodology usually requires more time and the allocation of specific resources. Moreover, as highlighted in a literature review conducted in April 2020 [40], such contributions—which are mostly feedback from experience or from the field—do not make it possible to assess the impact of the reported early adaptations, both in terms of SARS-CoV-2 transmission and of the effectiveness of psychiatric care in these new configurations [40]. In addition, the authors of feedback articles may have overestimated the potential of initiatives that have been implemented in their own work contexts, or to which they themselves have contributed. The articles included in the literature review are nonetheless of undeniable interest in that they provide the first keys to understanding the unprecedented situation in which the fields of psychiatry and mental health care have found themselves at the outset of the COVID-19 pandemic. Further, these articles show the willingness of those involved in psychiatry and mental health care to share organizational innovations and new professional practices implemented in response to the COVID-19 pandemic health crisis and reflect an acceleration of this trend on an international scale.

## 5. Conclusions

To sum up, this literature review highlights the need for pandemic preparedness, including in psychiatry and mental health services. Furthermore, although it illustrates how the COVID-19 pandemic has impacted psychiatric and mental health facilities and professionals globally in the early stages of the health crisis, it seems necessary to complete the analysis with individual and focus groups interviews and questionnaires to gather additional information. In this sense, it would be interesting to collect information from mental health professionals other than psychiatrists and psychologists—who are over-represented in this review—such as social workers, psychiatric nurses, occupational therapists, and professional peer helpers. It would also be interesting to ask them all about their personal experience of the changes reported in their working environments, processes, and conditions, in order to design methodologies and tools that could allow to improve professionals’ practices and quality of work life in psychiatric and mental health facilities. The final aims of the Psy-GIPO2C study—which is the framework of this literature review—is to formulate concrete recommendations for decision-makers to improve both working conditions and the quality of services provided in psychiatry and mental health services.

## Figures and Tables

**Table 1:** Search method of the literature review.

Database	Thesaurus	Search Terms
Pubmed (MEDLINE)	Yes	(“coronavirus”[Title/Abstract] OR “COVID-19”[Title/Abstract] OR “SARS-CoV-2”[Title/Abstract]) AND (“mental health worker”[Title/Abstract] OR “psychiatry”[Title/Abstract] OR “mental health professional”[Title/Abstract] OR “psychiatrist”[Title/Abstract] OR “psychologist”[Title/Abstract] OR “psychiatric nurse”[Title/Abstract] OR “e-professional in psychiatry”[Title/Abstract] OR “e-mental health” [Title/Abstract])
Cairn.info	No	(“COVID-19” OU “SARS-CoV-2” OU “coronavirus”) ET (“psychiatrie” OU “santé mentale” OU “psychologue” OU “infirmier en psychiatrie” OU “pair-aidant” OU “médiateur de santé pair” OU “e-professionnel de la psychiatrie”)
SantéPsy (Ascodocpsy)	Yes	[Ensemble de la base contient] (COVID-19) ET Mots-clés (“psychiatrie” OU “santé mentale” OU “psychologie” OU “hôpital psychiatrique”)

**Table 2:** Overview of the articles included.

Author(s), Date	Title	Methods	Aims	Results Relevant to Our Research	Country
Advenier F., Reca M.; 2020.	Teleconsultation in private practice during lockdown	Narrative literature review	To identify the difficulties of teleconsultation and the psychiatric symptomatology observed during the first lockdown during the COVID-19 pandemic.	Before the COVID-19 pandemic, teleconsultations were only used marginally as an alternative to face-to-face interviews. They were limited either to patients who had already been seen in person and had moved away or abroad, or to psychiatrists with a high level of digital literacy or who worked in locations with a low medical density. Most practitioners were reluctant to use digital technologies.	France; Europe
Al Joboory S., Monello F., Bouchard J.P.; 2020	PSYCOVID-19, psychological support device in the fields of mental health, somatic and medico-social	Feedback from field experience	To present the PSYCOVID-19 psychological support program that was developed and implemented in the context of the COVID-19 health crisis.	In the context of the COVID-19 health crisis, a psychological support program called “PSYCOVID-19” was developed. It is intended for the general population, and more than 241 mental health professionals have been involved in it.	France; Europe
Alavi Z., Haque R., Felzer-Kim I.T., Lewicki T., Haque A., Mormann M.; 2020.	Implementing COVID-19 Mitigation in the Community Mental Health Setting: March 2020 and Lessons Learned	Feedback from field experience	To share a plan of operations implemented in community mental health settings to ensure continuity of psychiatric care in the context of the COVID-19 pandemic.	In response to the COVID-19 pandemic, local community mental health service programs implemented a plan of operations that was aimed at mitigating the risk of SARS-CoV-2 transmission and developed using a Delphi process. Most routine appointments were transferred to phone or video assessments, except for patients for whom face-to-face appointments were absolutely necessary.	USA; America
Angelino A.F., Lyketsos C.G., Ahmed M.S., Potash J.B., Cullen B.A.; 2020.	Design and Implementation of a Regional Inpatient Psychiatry Unit for Patients who are Positive for Asymptomatic SARS-CoV-2	Feedback from field experience	To outline the decision process and ultimate design and implementation of a regional inpatient psychiatry unit for patients infected with asymptomatic SARS-CoV-2 and share key points for consideration in implementing future units elsewhere.	Faced with the COVID-19 pandemic, a regional inpatient psychiatry unit was established for patients with mental illnesses infected with asymptomatic SARS-CoV-2. As isolating asymptomatic patients is considered anti-therapeutic, the choice was made to preserve treatment methods as much as possible. The unit included a negative pressure area, a ventilation system, an airlock to put on PPE, etc. Telepsychiatry consultations were set up and games and activities were organized. One of the difficulties was to get patients to accept admission to this medical unit, the approach being not to impose it against their will.	USA; America
Anmella G., Arbelo N., Fico G., Murru A., Llach C.D., Madero S., et al.; 2020.	COVID-19 inpatients with psychiatric disorders: Real-world clinical recommendations from an expert team in consultation-liaison psychiatry	Feedback from field experience	To describe the impact of the COVID-19 pandemic on the work of consultation-liaison psychiatry teams, regarding drug prescription.	The combination of the most common first-line investigational therapies against COVID-19 (azithromycin, hydroxychloroquine, LPV/r and tocilizumab) involves several serious interactions that would normally contraindicate their co-administration. Patients with mental disorders, even those with complex psychopharmacological treatments, should not be excluded from receiving COVID-19 treatments. The benefit–risk assessment, which guides the recommendations, allows recommendations to be made.	International
Barry H., Doherty A.M., Clancy M., Moore S., MacHale S.; 2020.	Lockdown logistics in Consultation-Liaison Psychiatry	Feedback from field experience	To describe the adaptation of four Consultation–Liaison Psychiatry services to allow flexible and practical responses to the COVID-19 public health crisis.	In response to the COVID-19 health crisis, the four psychiatric consultation-liaison services rapidly set up telephone numbers—most of which can be reached 24/7—to identify needs and, on a case-by-case basis, refer to the right psychiatric or mental health service. On site, they developed triage systems and assessment areas.	Ireland; Europe
Bäuerle A., Graf J., Jansen C., Musche V., Schweda A., Hetkamp M., et al.; 2020.	E-mental health mindfulness-based and skills-based ‘CoPE It’ intervention to reduce psychological distress in times of COVID-19: study protocol for a bicentre longitudinal study	Study: evaluation of an intervention	To assess the efficacy of the e-mental health intervention ‘CoPE It’ in terms of reducing distress, depression and anxiety symptoms as well as improving self-efficacy, quality of life and mindfulness; and to evaluate the program’s usability, feasibility and participants’ satisfaction with ‘CoPE It’.	In the context of the COVID-19 health crisis, ‘CoPE It’ was developed as a low-threshold e-mental health program intended for people with mental disorders, aimed at reducing stress and anxiety. It is based on cognitive-behavioral therapy methods, and mindfulness exercises.	Germany; Europe
Bocher R., Jansen C., Gayet P., Gorwood P., Laprévote V.; 2020.	Responsiveness andsustainability of psychiatric care in France during COVID-19 epidemic	Narrative literature review	To examine the adaptations of French psychiatric care during the COVID-19 pandemic.	In response to the COVID-19 pandemic, French psychiatric facilities have adapted to mitigate the risk of transmission of SARS-CoV-2. They set up quarantine areas to admit new patients, developed psychiatric/Covid units dedicated to COVID-19-positive patients with mental disorders, and transferred outpatients to remote monitoring. In a psychiatric system that often remains hostile to digital technologies, this crisis has challenged mental health professionals into reshaping the therapeutic relationship to prevent patients from being lost to follow-up or relapsing.	France; Europe
Boland X., Dratcu L.; 2020.	COVID-19 and acute inpatient psychiatry: the shape of things to come	Feedback from field experience	To report on the experience of an English psychiatric unit during the first wave of the COVID-19 pandemic.	In order to deal with this health crisis, a variety of operational, inexpensive, and easy-to-implement responses were deployed: Everyday preventive actions, use of appropriate protective equipment, ventilation of closed spaces, regular team meetings to brief the unit’s nursing staff—who were exposed to a ‘tsunami’ of information about COVID-19—information posters in common areas, use of digital technologies to communicate with the outside, patient’s information and education, testing and isolation of patients tested positive for SARS-CoV-2, priority discharge of some patients, prohibition of visits, etc.	UK; Europe
Cabrera M.A., Karamsetty L., Simpson S.A.; 2020.	Coronavirus and Its Implications for Psychiatry: A Rapid Review of the Early Literature	Narrative literature review	To provide a critical synthesis of the scientific literature on the pandemic’s implications for psychiatric practice.	In response to the COVID-19 pandemic, numerous interventions have been implemented in psychiatry. However, no studies have yet evaluated the effectiveness of the measures adopted. The main change has consisted of rapid and extensive use of telepsychiatry. In practice, mental health professionals, in particular, have been playing a strong role in supporting patients, collaborating with primary care providers, and volunteer for crisis support hotlines.	International
Campanella S., Arikan K., Babiloni C., Balconi M., Bertollo M., Betti V., et al.; 2020.	Special Report on the Impact of the COVID-19 Pandemic on Clinical EEG and Research and Consensus Recommendations for the Safe Use of EEG	Study: survey	To describe the impact of COVID-19 on the use of electroencephalography and make recommendations to facilitate the re-establishment of access to non-invasive brain stimulation in clinical psychiatric care and research during the COVID-19 pandemic.	Non-invasive brain stimulation (NIBS) is used for the treatment of many neurological and psychiatric disorders. As a face-to-face procedure, it has been very limited in all countries in the early stages of the COVID-19 pandemic, which had a strong impact on related research activities.	International
Carpiniello B., Tusconi M., Zanalda E., Di Sciascio G., Di Giannantonio M.; 2020.	Executive Committee of The Italian Society of Psychiatry. Psychiatry during the COVID-19 pandemic: a survey on mental health departments in Italy	Study: survey via online questionnaires and statistical analysis	To report data relating to the Italian mental health system during the first phase of the COVID-19 epidemic.	In response to the COVID-19 pandemic, various adaptations have been implemented within Italian Mental Health Departments, multi-professional units—including community mental health centers—residential facilities, and psychiatric wards in general hospitals. Most staff members in these facilities expressed safety concerns, presumably due to major issues in the supply of Personal Protective Equipment.	Italy; Europe
Cave J., Crews M.; 2020.	Rehabilitation During a Pandemic: Psychiatrists as First Responders?	Feedback from field experience	To describe how a Community Rehabilitation Psychiatry team has adapted its interventions in response to COVID-19, particularly regarding clozapine monitoring.	During the COVID-19 health crisis, the team developed a specific procedure for the remote assessment of their patients, many of whom live in residential facilities and face increased health risks. Some of them are taking drugs such as clozapine, which requires blood count monitoring. During the pandemic, such monitoring has been less frequent.	International
Chen S., Jones P.B., Underwood B.R., Moore A., Bullmore E.T., Banerjee S., et al.; 2020.	The early impact of COVID-19 on mental health and community physical health services and their patients’ mortality in Cambridgeshire and Peterborough, UK	Study: analysis of quantitative data	To measure and analyze the impact of the COVID-19 pandemic on different aspects of care for people living with psychiatric disorders during the first wave.	In the early stages of the pandemic, the activity of the psychiatric care facilities declined, with no compensatory ‘rebound’ in demand observed at the time the study was carried out. There was also a shift from face-to-face to remote care, and the number of involuntary admissions decreased.	UK; Europe
Chen J.A., Chung W.J., Young S.K., Tuttle M.C., Collins M.B., Darghouth S.L., et al.; 2020.	COVID-19 and telepsychiatry: Early outpatient experiences and implications for the future	Literature review	To review key changes implemented at the beginning of the COVID-19 health crisis and helped usher in an unprecedented transformation in psychiatric care delivery, from mostly in-person to mostly virtual.	During the COVID-19 pandemic, the unprecedented use of telepsychiatry was made possible, in particular, by a relaxing of prior regulations for reimbursement of telepsychiatry services. Teleconsultations increased considerably, from under 5% virtual consultations to 97% in one year. Professionals have had to learn how to use new digital technologies, how to adapt their therapeutic relationships to virtual care, as well as how to organize new home-working settings and manage their own stress while continuing to provide adequate care for their patients.	USA; America
Cheng W., Zhang F., Hua Y., Yang Z., Liu J.; 2020.	Development of a psychological first-aid model in inpatients with COVID-19 in Wuhan, China	Feedback from field experience	To develop a mental health intervention model for inpatients that can be applied during a widespread epidemic, such as COVID-19.	During the COVID-19 health crisis, an onsite-online psychological first-aid model was developed, in which psychological healthcare workers from outside Wuhan were involved. An onsite psychiatrist coordinated the online work of the psychotherapists who had been recruited all over the country. A rapid assessment, triage and treatment process was developed, and the most serious cases were provided in-person care.	China; Asia
Chevance A., Gourion D., Hoertel N., Llorca P.M., Thomas P., Bocher R., et al.; 2020.	Ensuring mental health care during the SARS-CoV-2 epidemic in France: A narrative review	Narrative literature review	To propose guidance to ensure mental health care during the SARS-CoV-2 epidemic.	In the context of the SARS-CoV-2 pandemic, psychiatric patients are more vulnerable, due to a weakened immune system, frequent comorbidities, difficulties of health services to manage patients with mental disorders, etc. Psychiatric hospitals are more at risk of becoming transmission clusters not only due to community life, but also because their staff lack protective equipment and adequate training to deal with infectious diseases, because many professionals are exhausted, because it is harder for their patients to practice preventive actions, etc. In response to the pandemic, psychiatry has had to adapt, and it can play a role in coping with post-traumatic stress symptoms related to the pandemic itself.	France; Europe
Cohen D.; 2020.	Appreciating COVID-19 as a child and adolescent psychiatrist on the move	Feedback from personal experience	Report on his experience of the first wave of the COVID-19 pandemic in France as a child and adolescent psychiatrist. Describe how his hospital team reorganized to meet the new needs and outline the main emerging ethical issues.	During the COVID-19 health crisis, in this psychiatrist’s department, a unit was dedicated to autistic and behaviorally challenged children affected by COVID-19. In this unit, team members had two 3-h training courses on how to manage patients with COVID-19. More generally, all professionals working in the department were informed about the disease. Several ethical questions arose for this psychiatrist, especially about patients who had been identified as vulnerable and/or at risk of developing mental disorders in the context of this health crisis.	France; Europe
Columb D., Hussain R., O’Gara C.; 2020.	Addiction psychiatry and COVID-19: impact on patients and service provision	Feedback from field experience	To examine the impact of COVID-19 on addictions. To analyze the adaptations made in addiction services provision and how they could be an opportunity to improve the quality of services in the long term.	In response to the COVID-19 pandemic, adaptations have been made in the processes used in addiction psychiatry in Ireland, such as reducing the number of participants in focus groups or using digital platforms to hold remote group meetings. The introduction of new care procedures based on digital technologies may benefit patients in the long term, offering an opportunity to promote patient autonomy.	Ireland; Europe
Conrad R.C., Baum M.L., Shah S.B., Levy-Carrick N.C., Biswas J., Schmelzer N.A., et al.; 2020.	Duties toward Patients with Psychiatric Illness	Reflection	To give an overview of the various ethical issues that have arisen in psychiatry in this context, and to reflect on how to bring them together.	In the context of the pandemic, when a patient is admitted to a psychiatric hospital, they must be specifically protected from infection, as this is the case for people in nursing homes and prisons. While telepsychiatry may ensure continuity of care, its effectiveness for patients with mental health disorders has not yet been proven, and it may lead to social disparities in health. In the context of the COVID-19 health crisis, the state’s duties to its citizens living with mental health problems is being tested.	USA; America
Dursun O.B., Turan B., Pakyürek M., Tekin A.; 2020.	Integrating Telepsychiatric Services into the Conventional Systems for Psychiatric Support to Health Care Workers and Their Children During COVID-19 Pandemics: Results from A National Experience	Study	To present a program providing health care workers with psychosocial support, combining the use of telehealth applications with local psychosocial support teams, and determine its effectiveness.	Apart from the COVID-19 health crisis, it is difficult for health professionals to be attended by mental health services, for many reasons. The described system allows to provide them with psychological support, either for themselves or for their children, with an initial contact via a telehealth application, followed by a remote consultation with a psychiatrist.	Turkey; international.
El Hayek S., Cheaito M.A., Nofal M., Abdelrahman D., Adra A., Al Shamli S., et al.; 2020.	Geriatric Mental Health and COVID-19: An Eye-Opener to the Situation of the Arab Countries in the Middle East and North Africa Region	Study: literature review and distribution of questionnaires	To describe the impact of the COVID-19 pandemic on the field of geriatric psychiatry in the Arab countries of the Middle East and North Africa Region, during the early stages of the health crisis.	During the COVID-19 pandemic, interventions were implemented in the MENA region to support the elderly with psychiatric disorders: dedicated hotline numbers, dissemination of information, webinars, telepsychiatry, etc. However, such methods are not always appropriate for the geriatric population. There is a need to increase the allocation of adequate resources for geriatric mental health.	Egypt, Lebanon, Saudi Arabia, United Arab Emirates; international
Esterwood E., Saeed S.A.; 2020.	Past Epidemics, Natural Disasters, COVID19, and Mental Health: Learning from History as we Deal with the Present and Prepare for the Future	Literature review	To describe the effects of epidemics and natural disasters on mental health, in order to predict the impact of COVID-19 on mental health and propose strategies to best manage psychiatric symptoms and respond to increased needs.	The psychological consequences of a crisis such as the COVID-19 health crisis can range from the exacerbation of pre-existing disorders to stress or post-traumatic stress disorders. Front- and second-line health care workers may experience trauma and acute stress. Past experiences show that the consequences on mental health can be long-lasting. The psychological impact of quarantine in past pandemics has been revealed by post-traumatic stress symptoms, confusion, and anger. Promoting telehealth and online resources will be necessary to meet the growing demand resulting from the COVID-19 pandemic with a limited number of mental health professionals.	International
Fahed M., Barron G.C., Steffens D.C.; 2020.	Ethical and Logistical Considerations of Caring for Older Adults on Inpatient Psychiatry During the COVID-19 Pandemic	Feedback from field experience	To review measures taken to reduce the risk of transmission of COVID-19 and improve screening for infection in older adults.	During the COVID-19 pandemic, ethical challenges arise—for example, when a Covid-positive psychiatric patient refuses to wear a mask, when isolation in his room results in an increased frequency of episodes of agitation, or when another patient refuses to consent to testing.	USA; America
Fegert J.M. and Schulze U.M.E.; 2020.	COVID-19 and its impact on child and adolescent psychiatry—a German and personal perspective	Feedback from field experience	To report on the impact of the COVID-19 pandemic in the areas of child and adolescent psychiatry during the first wave.	In the early stages of the pandemic, in March and April 2020, it was the ‘calm before the storm’. Throughout the pandemic, child telepsychiatry, in particular, has grown considerably, and the health care system has recognized and adapted to these new forms of service provision in a short space of time.	Germany; Europe
Fovet T., Lancelevée C., Eck M., Scouflaire T., Bécache E., Dandelot D., et al.; 2020.	Mental health care in French correctional facilities during the COVID-19 pandemic	Literature review	To describe the reorganization of psychiatric care in French prisons in the context of the COVID-19 pandemic and examine the consequences of lockdown measures and early releases on inmates’ mental health.	During the COVID-19 pandemic, the French Prison Health Units and Regional Medico-Psychological Services, which respectively provide outpatient psychiatric care and day hospitalization in prisons, have been forced to adapt their practices substantially. To limit the risk of transmission of COVID-19, the prison administration has implemented various measures: The creation of COVID-19 areas, limitation of activities and visiting hours, early releases, etc.	France; Europe
Gautam M., Thakrar A., Akinyemi E., Mahr G.; 2020.	Current and Future Challenges in the Delivery of Mental Healthcare during COVID-19	Systematic literature review	To describe the impact of the COVID-19 pandemic on the mental health of health professionals, people with pre-existing mental health conditions, and the general population, and to describe future challenges.	In a pandemic such as COVID-19, psychiatric hospitals would be at risk of becoming transmission clusters, not only because of the large number of people gathered in one place, but also because some of these people, due to severe psychiatric symptoms, would not be able to understand the concept of social distancing, or because many are first admitted in emergency departments, increasing the risk of infection. Inpatient psychiatric services have imposed increasingly strict quarantine measures, while day hospitals and outpatient facilities have implemented remote monitoring.	USA and others; international
Green A.S., Ruchman S.G., Katz C.L., Singer E.K.; 2020.	Piloting forensic tele-mental health evaluations of asylum seekers	Feedback from field experience	To present human rights program which coordinated free forensic assessments by telephone or video asylum seekers from September 2019 to May 2020.	The remote forensic services offered by this program have been a relevant solution for individuals in immigration detention, particularly during the COVID-19 pandemic. However, some programs had to suspend forensic services because of the health crisis. This example could help them to maintain their services using telehealth.	USA and Mexico; international
Guan I., Kirwan N., Beder M., Levy M., Law S.; 2020.	Adaptations and Innovations to Minimize Service Disruption for Patients with Severe Mental Illness during COVID-19: Perspectives and Reflections from an Assertive Community Psychiatry Program	Feedback from field experience	To describe and reflect the adaptations and innovations experienced in a community psychiatry program.	Following the outbreak of the COVID-19 pandemic, the program arranged new office shifts, replaced some visits by telephone contact, increased communication and reliance on information from patients’ family members and housing workers, and delegated many medication deliveries to local pharmacies. Priority was given to ensure continuity of care for the most vulnerable patients. Moreover, the pandemic has been testing the resilience of healthcare providers. To promote coping in healthcare staff, the team tried to implement some principles: Breaking problems into smaller parts to solve; accepting emotions without judging them, staying healthy in day-to-day sleep and exercises; being mindful about values that reinforce the meaning of professional activity, and accepting personal limits.	Canada; America
Gulati G., Kelly B.D.; 2020.	Domestic violence against women and the COVID-19 pandemic: What is the role of psychiatry?	Narrative literature review	To determine the role that psychiatric services can play in addressing issues related to a heightened risk of domestic violence associated with mitigation measures taken during the COVID-19 pandemic.	In the context of the COVID-19 pandemic, rates of referral to mental health and psychological services have decreased, despite a likely increase in psychological distress, and mental illness. These trends are consistent with the experience of previous pandemics.	International
Han R.H., Schmidt M.N., Waits W.M., Bell A.K.C., Miller T.L.; 2020.	Planning for Mental Health Needs During COVID-19	Literature review	To review data on mental health sequelae from the 21st century pandemics, including SARS-CoV2, and offer explanations for observed trends, insights regarding anticipated needs, and recommendations for preliminary planning on how to best allocate limited mental health resources.	The data suggest that the mental health sequelae of the COVID-19 pandemic will be similar to those observed in the general population following other epidemics: Post-traumatic stress, depression, and suicide. Predictions regarding its economic impact suggest that depression and suicide rates may increase over time. Increased demand for psychiatric care due to a “post-pandemic mental health tsunami” is to be anticipated.	USA; America
Hou R., Yang L., Tang Z., Chen T.; 2020.	Caring for patients in mental health services during COVID-19 outbreak in China	Feedback from field experience	To reflect on some radical changes made in Chinese mental health services. To provide a reference to the effective delivery of mental health services in other countries through this pandemic.	In response to COVID-19, many mitigation measures have been taken in mental health services in China, including physical distancing; requiring everybody to wear adequate masks, as well as personal protective equipment depending on the risk; an initial on-site triage of patients and staff using temperature checks and recording travel and contact histories; a special admission procedure, with a two-week isolation period, regular testing for health care workers, a reorganization of teams and schedules; the use of WeChat application to communicate between staff members; a partial switch of psychiatric care to online services; or a 24/7 hotline aimed at promoting wellness in the general population.	China; Asia
Hoyer C., Ebert A., Szabo K., Platten M., Meyer-Lindenberg A., Kranaster L.; 2020.	Decreased utilization of mental health emergency service during the COVID-19 pandemic	Study: retrospective study	To assess the dynamics of mental health emergency service utilization rates during the COVID-19 pandemic.	During the COVID-19 pandemic, although the adoption of mitigating measures ensured the continuity of mental health services, the numbers of patients presenting to emergency departments. Quarantine and social distancing are expected to result in increased mental health disorders, as they did during the previous societal crisis.	Germany; Europe
Hsu S.T., Chou L.S., Chou F.H., Hsieh K.Y., Chen C.L., Lu W.C., et al.; 2020.	Challenge and strategies of infection control in psychiatric hospitals during biological disasters-From SARS to COVID-19 in Taiwan	Feedback from field experience	To present strategies that psychiatric hospitals can implement to prevent nosocomial infections among patients and staff, for example during an epidemic like the COVID-19 pandemic.	In Taiwan, in the context of COVID-19, the procedures that emerged from the lessons of the SARS epidemic were quickly implemented and adapted, with the help of the infection control committees within facilities. Online courses were provided to all staff members to improve their understanding of COVID-19 transmission and control. Social distancing was encouraged between staff members, with the division of staff into groups and the use of applications for online meetings. Most psychiatric hospitals have space arrangement plans in the event that people patients also have infectious diseases. The environment is set up to support hygienic behaviors (visual guidance, hooks for hanging clean gowns, etc.)	China; Asia
Ingram D.H., Best K.; 2020.	The Psychodynamic Psychiatrist and Psychiatric Care in the Era of COVID-19	Feedback from personal experiences	To document the impact of the COVID-19 pandemic on psychiatric and psychoanalytic work, practice, patients, and professionals during the first wave in the US.	During the COVID-19 pandemic, the number of new inpatient admissions initially dropped to about a quarter of the usual level. While the number of patients decreased, the epidemic brought new needs that the Department of Psychiatry could meet. In particular, support teams of mental health professionals and students enabled colleagues to do more intensive specialist work. After being briefed on each patient, they would call their family members to share information with them, receive their questions, share those with the team, and, if necessary, interact with the family. The pandemic led to an almost immediate restructuring of clinical care, with rapid and extensive use of telepsychiatry.	USA; America
Janeway D.; 2020.	The Role of Psychiatry in Treating Burnout Among Nurses During the COVID-19 Pandemic	Literature review	To describe the impact of the COVID-19 pandemic on the prevalence of burnout among US nurses, and the measures taken to prevent it.	Apart from COVID-19, consultation-liaison psychiatry includes the creation of programs that usually take the form of interdisciplinary meetings with professionals from other medical-surgical departments who provide support and guidance when working with the most challenging, high-risk patients. Consultation-liaison psychiatry and employee assistance programs make sense in the context of the COVID-19 pandemic, as they can bring innovative solutions to reduce significantly stress levels and help to prevent burnout.	USA; America
Kavoor A.R., Chakravarthy K., John T.; 2020.	Remote consultations in the era of COVID-19 pandemic: Preliminary experience in a regional Australian public acute mental health care setting	Feedback from field experience	To describe how a setting dealing with acute mental health problems has adapted to the COVID-19 pandemic.	In response to the COVID-19 pandemic, several changes have been implemented in this acute mental health care service to maintain a balance between limiting the risk of SARS-CoV-2 transmission and ensuring continuity of care.	Australia
Kelly B.D.; 2020.	Emergency mental health legislation in response to the COVID-19 (Coronavirus) pandemic in Ireland: Urgency, necessity and proportionality	Literature review	To present the content of the Irish 2001 mental health legislation, as well as the emergency legislation that was adopted in response to the COVID-19 pandemic to address mental health issues. To examine whether the measures taken and implemented were indeed urgent, necessary, and proportionate.	Before the COVID-19 pandemic, Ireland’s Mental Health Act was passed in 2001 and fully commenced in 2006. The Emergency Measures in the Public Interest (COVID-19) Act was passed by the Irish Parliament and signed by the Irish President on 27 March 2020 and the provisions related to mental health came into effect on 30 March 2020. This Act amends the legislation previously in force regarding the definition of certain terms, independent psychiatric reports and mental health tribunals.	Ireland; Europe
Kelly B.D.; 2020.	Plagues, pandemics and epidemics in Irish history prior to COVID-19 (coronavirus): what can we learn?	Reflection	To provide a brief overview of epidemics and pandemics in Irish history. To identify any lessons that might be useful in psychiatry in the context of COVID-19.	Prior to the COVID-19 pandemic, various epidemics hit Ireland. Some particularly impacted large, unhygienic mental hospitals. While public health responses have evolved, psychological effects to epidemics are comparable—ranging from moderate to excessive anxiety, including panic—and can be long-lasting. In addition to the management of the illness caused by the virus, these reactions are an issue in themselves and must be anticipated.	Ireland; Europe
Kennedy H.G., Mohan D., Davoren M.; 2020.	Forensic psychiatry and COVID-19: accelerating transformation in forensic psychiatry	Feedback from field experience	To describe the impact of the COVID-19 pandemic on forensic psychiatry services, including prisons, in Ireland.	In the first few weeks of the COVID-19 pandemic, as firefighting became the rule, mental health professionals had to quickly reassess how to practice safely and effectively from day to day. The switch to remote working by phone and video improved efficiencies and generated new risks. In forensic psychiatry, court appearances of patients were transferred to “telepresence”, which seems to have been very beneficial, as, for example, video-linked court appearances reduce the risk of escape or abscond, further minimizing the use of restrictive practices such as handcuffs.	Ireland; Europe
Khanna R., Murnane T., Kumar S., Rolfe T., Dimitrieski S., McKeown M., et al.; 2020.	Making working from home work: reflections on adapting to change	Feedback from field experience	To report on the experience of a mental health unit whose professionals worked from home.	In response to the COVID-19 pandemic, the use of telepsychiatry, which had been marginal until then, has become more widespread. The shift from face-to-face to remote consultations went along with a shift to telework. Over this period—compared to the same period in the previous year—and including these remote consultations, there were 3% more consultations and 7% fewer cancellations or non-participation in consultations.	Australia
Korsnes M.S., Grødal E., Kjellén E., Kaspersen T.M.C., Gjellesvik K.B., Benth J., et al.; 2020.	COVID-19 Concerns Among Old Age Psychiatric In- and Out-Patients and the Employees Caring for Them, a Preliminary Study	Exploratory study: survey via questionnaires among users and professionals	To investigate the impacts of the COVID-19 pandemic on the quality of psychiatric care and life of senior mental health services users during the first wave in Norway, and on the working conditions of professionals working with them.	Due to the COVID-19 health crisis, among those professionals working with senior mental health service users, some shared concerns about the risk of unintentionally infecting one of their patients, particularly because of the existence of asymptomatic forms of the disease; others complained about general stress due to the health crisis context; a few criticized the way the crisis had been handled; and one spoke of his fear of being infected by SARS-CoV-2. Overall, most of them responded that their working conditions had been negatively impacted by this health crisis.	Norway; Europe
Kreuzer P.M., Baghai T.C., Rupprecht R., Wittmann M., Steffling D., Ziereis M., et al.; 2020.	SARS-CoV-2 Risk Management in Clinical Psychiatry: A Few Considerations on How to Deal With an Unrivaled Threat	Feedback from field experience	To exemplarily describe elements related to the internal risk management, the organizational and structural changes, and the communicational strategies applied in response to the COVID-19 pandemic in a psychiatric hospital in Southern Germany.	Faced with an epidemic such as that of COVID-19, it has been difficult for psychiatry departments to implement strict infection control, as psychiatric care involves numerous contacts, particularly due to collective activities. In the said hospital, in the early stages of the pandemic, outpatient treatment facilities were shut down for several weeks and the number of inpatients was reduced. All inter-sectoral activities were closed; visits were limited; screening procedures were drawn up; suspected positive cases have been admitted to psychiatric isolation units: A ‘traffic light’ zone concept. Some challenges emerged, such as a lack of human resources, the impossibility to take holiday leaves, causing burnouts. Some professionals were also quickly reassigned, which required rapid adaptation.	Germany; Europe
Lecoquierre A., Diarra H., Abed N., Devouche E., Apter G.; 2020.	Expérience d’une plateforme d’écoute psychologique multilingue nationale durant le confinement dû à la COVID-19	Feedback from field experience	To present a national multilingual psychological support platform, deployed in France during the first wave of the COVID-19 pandemic.	In the context of the COVID-19 health crisis, a crisis unit was set up, first locally, before being extended throughout the country. It consists of a multilingual telephone platform, to which many professionals from mental health services contributed on a voluntary basis to offer emergency listening and psychological support. This platform aims to identify people in psychological distress and to prevent their admissions to emergency departments of hospitals.	France; Europe
Looi J.C., Allison S., Bastiampillai T., Pring W.; 2020.	Private practice metropolitan telepsychiatry in larger Australian states during the COVID-19 pandemic: an analysis of the first 2 months of new MBS telehealth item psychiatrist services	Study: analysis of quantitative data	To investigate the uptake of video and telephone telehealth consultations in April-May 2020, and the overall changing rates of consultation, across the larger states of Australia.	During the COVID-19 pandemic, remote monitoring of psychiatric patients was largely used. Telephone and video were used more for short consultations (15 to 30 min) and long consultations (30 to 75 min), respectively. Overall, the telephone was used more than the video.	Australia
Lyne J., Roche E., Kamali M., Feeney L.; 2020.	COVID-19 from the perspective of urban and rural general adult mental health services	Feedback from field experience	To describe the impact of the COVID-19 pandemic on German and Irish general adult mental health services.	In the early stages of the COVID-19 pandemic, the said mental health units responded to the needs identified and developed a consistent plan for service delivery. In practice, for staff, this meant taking steps such as avoiding shared offices, working from home whenever possible, replacing face-to-face meetings with video conferencing, telephone or email contact, and using telemedicine devices.	Germany and Ireland; international
Ma J., Zhong H., Jiang M., Zeng K., Zhong B., Liu L., et al.; 2020.	Emergency response strategy for containing COVID-19 within a psychiatric specialty hospital in the epicenter of the COVID-19 epidemic in China	Feedback from personal experiences	To present the accumulated experience of the authors during the process of combating COVID-19 in a psychiatric hospital. To provide a reference for psychiatric specialty hospitals and institutions that treat large populations of chronically ill patients in other parts of the world.	In response to the COVID-19 pandemic, this hospital developed a plan consisting of reorganizing services, optimizing resource allocation (staff and protective equipment), complying with various procedures (particularly admission procedures), and preventing nosocomial diseases.	China; Asia
Marehin M.S., Mboumba Hinnouo A., Obiang P.A.; 2020.	Organization of psychiatric care in Gabon during the COVID-19 epidemic	Literature review	To describe the impact of the COVID-19 pandemic on the field of psychiatry in Gabon during the early stages of the health crisis.	During the COVID-19 pandemic, the main mental health facilities were left out of the government’s measures, and professionals working in these facilities had access to few or no protective equipment, for example. The health crisis has intensified the dysfunctions observed in psychiatric care.	Gabon; Africa
McGrath J.; 2020.	ADHD and COVID-19: current roadblocks and future opportunities	Feedback from field experience	To examine the impact of the COVID-19 pandemic on the psychiatric care of young people with attention-deficit hyperactivity disorder.	In the context of the COVID-19 epidemic, psychiatric care for children with ADHD had to adapt. In particular, digital consultations have been reshaping the assessment and treatment of those patients.	Ireland; Europe
Mellins C.A., Mayer LES, Glasofer D.R., Devlin M.J., Albano A.M., Nash S.S., et al.; 2020.	Supporting the well-being of health care providers during the COVID-19 pandemic: The ‘CopeColumbia’ response	Feedback from field experience	To describe ‘CopeColumbia’, a peer support program developed by a faculty in a large urban medical center’s Department of Psychiatry to support emotional well-being and enhance the professional resilience of health care workers.	In the context of the COVID-19 health crisis, a program was developed with the following objectives: To promote mental health and prevent the onset of psychological disorders such as stress or depression among health professionals, and to identify possible needs for more formal psychiatric care. Three categories of services were developed: Peer support groups, one-to-one sessions, and conferences on different topics (stress, anxiety, trauma, loss and bereavement, etc.). All resources have been made available 24/7 on a dedicated website.	USA; America
Naarding P., Oude Voshaar R.C., Marijnissen R.M.; 2020.	COVID-19: Clinical Challenges in Dutch Geriatric Psychiatry	Feedback from field experience	To describe the impact of the COVID-19 pandemic on geriatric psychiatry during the first wave in the Netherlands.	In response to the COVID-19 pandemic, measures were rapidly taken to adapt to the context. In addition to mitigation measures taken to prevent the spread of the virus, a switch to mobile and e-health services was implemented. Specific COVID-19 isolation units have been set up in most mental health centers. Geriatric psychiatry teams have faced many clinical challenges, as well as ethical issues. For example, denying family visits even to those who are dying has raised questions, as it means not allowing a proper farewell period and induces a risk of developing pathological grief.	The Netherlands; Europe
Nabe-Nielsen K., Nilsson C.J., Juul-Madsen M., Bredal C., Hansen L.O.P., Hansen Å M.; 2020.	COVID-19 risk management at the workplace, fear of infection and fear of transmission of infection among frontline employees	Study: survey via electronic questionnaires	To describe and compare COVID-19 risk management among frontline staff working in geriatric care, hospital/rehabilitation, psychiatry, childcare and ambulance services To document COVID-19 the association of risk management with fear of infection and fear of infection transmission.	When confronted with the COVID-19 pandemic, 30% to 49% of health professionals reported that they feared being infected at work, with the highest percentages among ambulance services and geriatric care workers, and the lowest among employees in psychiatry.	Denmark; Europe.
Normand M.; 2020.	A short chronicle of psychiatry in the time of COVID-19	Study: interview survey with psychologists	To describe the impact of the COVID-19 pandemic on psychiatry in France during the first wave.	The survey revealed that, during the early COVID-19 health crisis, there was no increased demand for consultations or hospital admissions in psychiatric care. During the first period, a new temporality appeared, with lower rates than those usually observed.	France; Europe
Paul E., Crommelinck B., Decker M., Doeraene S., Kaisin P., Lallemand B., et al.; 2020.	The impact of the COVID-19 crisis on a child and adolescent psychiatric hospital	Feedback from field experience	To describe the impact of the COVID-19 pandemic on a French child psychiatry facility during the first wave.	During the COVID-19 pandemic, psychiatric care units have been reorganized and team dynamics have been disrupted. Therapeutic care based on the dynamics of going back and forth between the family and the institution has been challenged. Distancing has replaced proximity, which is the norm in child psychiatry. Welcoming gestures, and demonstrations of affection, consolation, reassurance, and restraint have turned into preventive gestures, with smiles hidden behind face masks. Everyone was led to leave their comfort zone and to challenge their assumptions.	France; Europe
Pignon B., Gourevitch R., Tebeka S., Dubertret C., Cardot H., Dauriac-Le Masson V., et al.; 2020.	Dramatic reduction of psychiatric emergency consultations during lockdown linked to COVID-19 in Paris and suburbs	Study: quantitative methods	To compare the numbers of and reasons for emergency psychiatric consultations in 3 psychiatric emergency departments in Paris or the Paris region during the first 4 weeks of lockdown related to the COVID-19 pandemic with those of the same period the year before the health crisis, in 2019.	During the COVID-19 pandemic, in the three psychiatric emergency departments, the same decreasing trend in the number of consultations was observed, with a total of 553 emergency psychiatric consultations during the first 4 weeks of lockdown, compared to 1224 during the same period in the previous year, i.e., a 54.8% drop. In the three units, this decrease was observed for all psychiatric diagnoses and reasons, including suicide attempts. However, the number of involuntary admissions increased.	France; Europe
Ping N.P.T., Shoesmith W.D., James S., Nor Hadi N.M., Yau E.K.B., Lin L.J.; 2020.	Ultra Brief Psychological Interventions for COVID-19 Pandemic: Introduction of a Locally Adapted Brief Intervention for Mental Health and Psychosocial Support Service	Feedback from field experience	To present how ultra-brief psychological interventions (UBPI) were adapted and used with healthcare providers dealing with COVID-19, as well as with the public who required psychological first aid.	During the COVID-19 pandemic, the UBPI was used in different ways: a self-guided, peer-supported intervention was developed for mental health at work; and the ‘COVIDCare’ online chat platform was set up to provide psychological support to anyone suffering from stress or anxiety, to strengthen the skills of the staff responsible for attending on people online.	Malaysia; Asia
Roncero C., García-Ullán L., de la Iglesia-Larrad J.I., Martín C., Andrés P., Ojeda A., et al.; 2020.	The response of the mental health network of the Salamanca area to the COVID-19 pandemic: The role of the telemedicine	Study: observational study	To report on the impact of the COVID-19 pandemic on mental health services in Spain, and how a local mental health network responded.	During the first 4 weeks of the COVID-19 pandemic, admission numbers were 65% lower than during the same period of the previous year, and 37% lower during the following 4 weeks. The Mental Health Network of the Salamanca area reorganized its resources within a few weeks: They closed some units, opened a specific unit for patients with COVID-19, and reorganized human resources. They also implemented telepsychiatry and specific programs: One was designed to promote mental health among psychiatric patients and professionals; the other was designed to provide support to homeless people.	Spain; Europe
Rosen B., Preisman M., Hunter J., Maunder R.; 2020.	Applying Psychotherapeutic Principles to Bolster Resilience Among Health Care Workers During the COVID-19 Pandemic	Feedback from field experience	To discuss the development of a resilience coaching model developed in a Toronto hospital that is rooted in principles from psychotherapeutic literature and practice to support psychological well-being among hospital-based health care workers.	In the context of a health crisis, building the resilience of health workers can improve their well-being and enable them to continue to work in the conditions required for their job, thus helping to maintain the overall functioning of the health care system. In response to the COVID-19 health crisis, a “resilience coaching” program, which had been created in the wake of the SARS epidemic was developed for hospital-based health care workers to bolster their resilience during a pandemic.	Canada; America
Rovers J.J.E., van de Linde L.S., Kenters N., Bisseling E.M., Nieuwenhuijse D.F., Oude Munnink B.B., et al.; 2020.	Why psychiatry is different—challenges and difficulties in managing a nosocomial outbreak of coronavirus disease (COVID-19) in hospital care	Study: case study, distribution of questionnaires	To determine which psychiatry-specific factors contributed to a nosocomial outbreak that occurred in a psychiatric department and provide possible interventions in future outbreak management.	During the first wave of the COVID-19 pandemic, a cluster emerged in a psychiatric facility in the Netherlands: 19% of the patients and 43% of the health care workers were infected with SARS-CoV-2. Several factors specific to psychiatry have been identified: Severe psychiatric patients are more at risk for lung disease and depressed psychiatric patients are more at risk for infectious diseases in general; severe psychiatric patients are often unaware of their physical symptoms due to cognitive deficits or reduced sensitivity to pain, and often have trouble communicating; recognition and management of physical disease in psychiatric patients is suboptimal compared to the general population; psychiatrists generally consider that their primary is to manage mental health disorders and may overlook signs of physical disease; psychiatric medications tend to negatively influence patients’ ability to understand and follow instructions; psychiatric patients often engage in group activities and psychiatric care units are organized around community life.	The Netherlands; Europe
Roy A., Singh A.K., Mishra S., Chinnadurai A., Mitra A., Bakshi O.; 2020.	Mental health implications of COVID-19 pandemic and its response in India	Literature review	To review the prevailing mental health issues during the COVID-19 pandemic through global experiences, and reactive strategies established in mental health care.	Although Indian mental health services have adapted in response to the COVID-19 pandemic, there has been less penetration of digital mental health solutions. In a context where a large part of the population is vulnerable and where misinformation is omnipresent on social media, a hotline has been set up and mental health education messages have been disseminated.	India; Asia
Russ M.J., Sisti D., Wilner P.J.; 2020.	When patients refuse COVID-19 testing, quarantine, and social distancing in inpatient psychiatry: clinical and ethical challenges	Reflection	To discuss the new ethical challenges in the care of patients with serious psychiatric illness who require inpatient treatment and who may have been exposed to COVID-19 or have mild to moderate COVID-19 but refuse testing and adherence to infection prevention protocols.	During epidemics like the COVID-19 one, the risk of transmission of the virus is particularly high in psychiatric hospitals. Professionals working in inpatient psychiatric care have been facing a new dilemma: To respect the patient’s autonomy and restore their capacity while also mitigating infection risk to themselves and others. As every effort must be made to engage in shared decision-making, regulatory guidelines for dealing with a patient’s refusal of testing or treatment have not yet been established.	USA; America
Scharf D., Oinonen K.; 2020.	Ontario’s response to COVID-19 shows that mental health providers must be integrated into provincial public health insurance systems	Feedback from field experience	To describe the impact of the COVID-19 pandemic on the mental health of the population in Canada and describe Ontario’s state response to this mental health crisis.	Part of Ontario’s response to the mental health crisis caused by the COVID-19 health crisis has consisted of new Health Insurance Program billing codes, and subsequent reimbursement plans covering mental health care provided by physicians.	Canada; America
Shalev D., Nakagawa S., Stroeh O.M., Arbuckle M.R., Rendleman R., Blinderman C.D., et al.; 2020.	The Creation of a Psychiatry-Palliative Care Liaison Team: Using Psychiatrists to Extend Palliative Care Delivery and Access During the COVID-19 Crisis	Feedback from field experience	To present a model for rapid palliative care workforce expansion under crisis conditions, using supervised advanced psychiatry trainees to provide primary palliative services in the acute care and emergency setting.	The wave of COVID-19 infection led to an increase in the need for palliative care in New York City. A palliative care team dedicated to COVID-19 patients was set up but was quickly overwhelmed. Advanced psychiatry trainees were quickly trained and worked with the said unit. Although they had no specific skills, they possessed competencies in the areas of serious illness communication and psychosocial aspects of medical illness. This facilitated their rapid contribution to palliative care, as they could manage cases of delirium and agitation, and provide psychosocial support. They also facilitated video conference visits between patients and families.	USA; America
Shi Y., Wang J., Yang Y., Wang Z., Wang G., Hashimoto K., et al.; 2020.	Knowledge and attitudes of medical staff in Chinese psychiatric hospitals regarding COVID-19	Study: online questionnaire survey of psychiatric professionals, psychiatrists, and psychiatric nurses	To assess the knowledge and attitudes of medical staff in two Chinese mental health centers during the COVID-19 outbreak.	During the COVID-19 health crisis, 64% of the medical staff of the psychiatric hospitals studied have received the relevant training. 78% are confident in their level of knowledge to protect themselves from the risk of contamination with COVID-19. 77% expressed their willingness to care for COVID-19 positive patients. Although no statistical association was revealed between the willingness to care for infected patients and age, sex, or marital status, advance training and experience, and the confidence in one’s knowledge of risks and protection was associated with a greater likelihood of accepting to care for such patients.	China; Asia
Thome J., Coogan A.N., Simon F., Fischer M., Tucha O., Faltraco F., et al.; 2020.	The impact of the COVID-19 outbreak on the medico-legal and human rights of psychiatric patients	Reflection	To discuss the legal implications of the COVID-19 pandemic for psychiatry and report a novel situation in which psychiatric patients may experience a diminution of their statutory protections.	During this health crisis, in many countries, the rights of psychiatric professionals to use coercive methods have been expanded to enforce infection control measures. Fundamental values and principles need to be formalized to ensure effective protection of patients’ rights in mental health settings.	International
Thome J., Deloyer J., Coogan A.N., Bailey-Rodriguez D., da Cruz E.S.O.A.B., Faltraco F., et al.; 2020.	The impact of the early phase of the COVID-19 pandemic on mental health services in Europe	*Ad-hoc* survey conducted among 23 experts via questionnaires	To identify transnational challenges to which the COVID-19 pandemic confronts psychiatric patients and mental health services across Europe.	In the context of the COVID-19 health crisis, challenges have emerged for the national psychiatric and mental health sectors in Europe. They are related to the consequences on the mental health of the pandemic and the societal measures to combat it; the management of psychiatric problems in COVID-19 negative and positive patients; and the protection of mental health professionals ensuring continuity of care. The findings are the following: Health policies mostly overlook mental health and focus on the management of COVID-19; psychiatric professionals have mostly focused on inpatients, with the need to prevent transmission of the virus, while outpatient care has been more discontinuous; during the early phase of the epidemic, the use of psychiatric care decreased, but many patients deteriorated (psychotic decompensation, relapse, etc.)	International
Usman M., Fahy S.; 2020.	Coping with the COVID-19 crisis: an overview of service adaptation and challenges encountered by a rural Psychiatry of Later Life (POLL) team	Feedback from field experience	To highlight several clinical, administrative, medicolegal and IT implications of the COVID-19 pandemic on the delivery of mental healthcare to an elderly vulnerable patient cohort due to recommended social distancing measures.	In response to the COVID-19 pandemic, it was quickly decided to use telepsychiatry with those patients, as they are elderly and therefore more at risk. Continuity of care was ensured by telephone, or video calls. Professionals tried to propose activities to patients (relaxation, puzzles, crossword puzzles, TV shows, radio shows, etc.). Their work focused on maintaining patients’ well-being since, in this context and without in-person contact, discussing some topics such as past traumas could not be considered.	Ireland; Europe
Viswanathan R., Myers M.F., Fanous A.H.; 2020.	Support Groups and Individual Mental Health Care via Video Conferencing for Frontline Clinicians During the COVID-19 Pandemic	Feedback from field experience	To present how psychiatry departments can develop support systems to help frontline health care workers cope with the stress induced by the COVID-19 pandemic.	During the COVID-19 pandemic, peer support groups by videoconferencing and telephone have been developed for physicians and nurses, focusing on issues and emotions related to their frontline clinical work with COVID-19 patients. These groups are led by attending psychiatrists and psychiatric residents.	USA; America
Wasser T., Hauser L., Kapoor R.; 2020.	The Management of COVID-19 in Forensic Psychiatric Institutions	Feedback from field experience	To present the specific problems posed by COVID-19 in the field of forensic psychiatry.	During the COVID-19 pandemic, forensic psychiatric institutions have had to develop specific policies related to PPE, testing, and management of suspected and confirmed COVID-19 cases. Specific guidance is needed in forensic psychiatric institutions because their framework differs substantially from that of both acute-care psychiatric hospitals and correctional institutions. Forensic hospitals have had to impose restrictions on visits not only from family and friends, but also from professional such as lawyers. To address this concern, they have promoted the use of video communication. Any change in patients’ treatment plans requires the approval of the judicial administration, which has been very difficult to obtain in the context of the pandemic. Early discharges have not always been planned and coordinated, which has posed problems regarding continuity of psychiatric care.	USA; America
Wulfman R., Jourdain P., Ourahou O.; 2020.	During trauma: An approach to the psychiatric pathology of COVID-19 patients through the COVIDOM platform	Feedback from field experience	To present ‘Covidom’, a French e-mental health application that has been implemented during the COVID-19 health crisis.	In the context of the COVID-19 health crisis, ‘Covidom’ was developed with the voluntary help of mental health trainees or experienced. Its aim is to allow psychiatric patients with (or suspected of having) COVID-19 without severe symptoms to benefit from remote monitoring. Other health professionals have also benefited from this program.	France; Europe
Xiang Y.T., Zhao Y.J., Liu Z.H., Li X.H., Zhao N., Cheung T., et al.; 2020.	The COVID-19 outbreak and psychiatric hospitals in China: managing challenges through mental health service reform	Feedback from field experience	To outline major challenges for patients with psychiatric disorders and mental health professionals during the COVID-19 outbreak, and to discuss how to manage these challenges through further mental health service reform in China.	Due to the strict quarantine measures imposed in China during the COVID-19 health crisis, patients who have suspected or confirmed COVID-19 are at high risk of developing mental health problems. In response, crisis psychological intervention teams were established in many psychiatric hospitals. Psychiatric hospitals are vulnerable to outbreaks. In addition, frequent overcrowding and group activities increase the risk of transmission of COVID-19 in psychiatric hospitals. As an alternative, isolation wards have been set up in psychiatric hospitals for patients with suspected and confirmed COVID-19.	China; Asia
Zhang E., LeQuesne E., Fichtel K., Ginsberg D., Frankle W.G.; 2020.	In-patient psychiatry management of COVID-19: rates of asymptomatic infection and on-unit transmission	Process improvement and quality improvement study	To detail the response of the in-patient psychiatric services of a New York City-based facility to the COVID-19 outbreak from 1 March to 1 May 2020.	With the COVID-19 pandemic, in-patient psychiatric units have made rapid changes: Patients have had to wear adequate masks in the common areas; hand sanitizer dispensers were mounted on the walls; ‘high-touch’ surfaces have been cleaned frequently; meals have been delivered and medications distributed room-to-room; face-to-face group therapies were gradually eliminated; no visitors were allowed on the in-patient psychiatry units; the electroconvulsive therapy service was temporarily closed. To mitigate the adverse impact of these measures, some patients were provided with individual electronic devices with streaming entertainment services and newspapers and could use technology to video conference with family and friends. All staff meetings were switched to video conference as well. Some of the staff, including administrative staff, as well as NYU medical students, were pulled from the unit. During the first wave, the number of admissions to the in-patient psychiatric units dropped by 22% compared to the same time period in 2019.	USA; America

**Table 3:** Information about the selected articles.

Characteristics	All Articles (*n* = 72)
**Area of study**	
Europa	30 [7,13,16,17,18,19,20,21,22,23,24,25,26,27,28,29,30,31,32,33,34,35,36,37,38,39,40,41,42,43]
America	17 [6,44,45,46,47,48,49,50,51,52,53,54,55,56,57,58,59]
Asia	8 [9,60,61,62,63,64,65,66]
Oceania	3 [67,68,69]
Africa	1 [70]
International	13 [5,8,39,40,57,71,72,73,74,75,76,77,78]
**Type of article**	
Experience feedback	38 [6,9,16,18,19,21,25,26,29,30,33,35,36,42,43,44,46,47,48,49,52,53,54,55,56,57,58,60,62,63,64,66,67,68,73,75,77,79]
Study	17 [5,17,20,23,27,28,34,37,38,41,59,65,69,71,74,76,80]
Literature review	13 [8,13,22,24,31,32,39,40,45,51,61,70,72]
Reflection	4 [7,50,78,81]
**Contributions to mental health promotion in the context of the COVID-19 health crisis**	
Towards the general population	7 [16,17,18,19,20,60,61]
Towards other health professionals	5 [44,45,46,47,71]
In other units and/or specialties	4 [21,48,49,62]
During and after the COVID-19 health crisis	13 [5,7,8,13,22,23,39,40,50,51,52,53,72]
**Organizational adaptations in and out psychiatric hospitals**	
In facilities	38 [9,13,20,21,22,23,25,26,27,28,29,30,31,33,35,36,38,39,41,42,43,49,52,54,55,57,58,59,63,64,65,66,67,70,76,77,79,80]
In private medical practices	3 [24,58,69]
Within the community	5 [6,27,56,58,75]
Related to somatic care	3 [35,59,74]
Related to drug prescription	4 [53,56,73,75]
**Evolution of regulations**	
Related to telepsychiatry	2 [52,58]
Related to involuntary psychiatric care	3 [32,78,81]
Related to involuntary testing	2 [55,81]
**Well-being at work and professional’s**	
personal feedbacks	15 [16,21,26,34,37,41,44,45,48,52,58,61,65,68,81]

## Data Availability

Data are fully available and will be shared upon request to J.G.B.
